# L'observance thérapeutique dans les dermatoses chroniques: à propos de 200 cas

**DOI:** 10.11604/pamj.2015.22.116.6094

**Published:** 2015-10-12

**Authors:** Nissrine Amraoui, Salim Gallouj, Mohamed Amine Berraho, Chakib Najjari, Fatima zohra Mernissi

**Affiliations:** 1 Service de Dermatologie CHU Hassan II, Fès, Maroc; 2 Service d'Epidemiologie et de Recherche Clinque, CHU Hassan II Fès, Maroc

**Keywords:** Observance thérapeutique, dermatose chronique, suivi, Compliance to treatment, chronic dermatosis, follow-up

## Abstract

**Introduction:**

L'observance thérapeutique est la capacité à prendre correctement son traitement, tel qu'il est prescrit par le médecin. Elle est peu étudiée en dermatologie.

**Méthodes:**

Le but de notre étude est d’évaluer l'observance chez les patients suivis au service de dermatologie du Centre Hospitalier Universitaire Hassan II de Fès pour une dermatose chronique et de rechercher les facteurs liés à une mauvaise observance à travers une étude incluant 200 patients suivis depuis au moins 6 mois. L’évaluation de l'observance s'est faite essentiellement à l'aide d'un entretien et les facteurs liés à l'observance ont été recherchés par un questionnaire.

**Résultats:**

68% de nos patients étaient observant, la mauvaise observance était associée à un niveau socio économique et d’étude bas, à une vie solitaire, à une durée de suivi longue, aux effets secondaires et au coût élevé des traitements, à l'absence d'efficacité, à une faible visibilité des lésions, à une ordonnance complexe, à une explication faible de la maladie, et à des difficultés d'accès à la consultation. Le taux d'observance retrouvé dans notre étude est un taux satisfaisant selon les données de la littérature, notre étude a confirmée certaines facteurs connues et a mis le point sur d'autres facteurs peu étudiés tel un traitement traditionnel associé, la part de chaque forme de traitement dans le respect de l'ordonnance, les dermatoses les plus touchées par les difficulté d'observance a savoir les dermatoses bulleuses et le psoriasis, et l'intérêt du pharmacien.

**Conclusion:**

Cette analyse de la fréquence de ce phénomène et des facteurs essentiels qui l'influencent permet de cibler la prise en charge à travers une personnalisation de l'entretien médical, une adaptation du suivi au contexte de nos patients et à la nature de notre institution de santé.

## Introduction

La peau est un organe privilégié de la vie de relation, son atteinte bouleverse l'image de l'individu envers lui-même et implique que devant une maladie cutanée, le patient s'acharne pour restaurer son image. Mais quand il s'agit d'une maladie cutanée chronique, cette volonté peut manquer pour différentes raisons et le suivi thérapeutique peut être semé d'obstacles. La compréhension des déterminants de l'observance médicamenteuse est incontournable dans l'objectif d'envisager des actions pour l'optimiser et ou le soutenir.

## Méthodes

Le but de l’étude est d’évaluer le niveau d'observance thérapeutique chez des patients suivis pour dermatose chronique en service de dermatologie et de vénérologie de centre hospitalier universitaire Hassan II de FES et déterminer les facteurs qui l'affectent afin d’établir une prise en charge adaptée au contexte de nos malades. Nous avons opté pour une étude transversale à recrutement prospectif réalisée au service de Dermatologie du centre hospitalier universitaire Hassan II de Fès, étalée sur une période de 9 mois incluant des patients suivis en consultation pour dermatose chronique évoluant depuis au moins 6 mois. Les critères d'exclusion étaient le refus du patient et l’âge moins de 12ans. nous avons fait appel afin de mesurer l'observance de nos malades, à des moyens directs, représentés par le comptage des comprimés restants et des conditionnements et l'observation des effets clinques des traitement, qu'on a associé à d'autres moyens indirects qui sont essentiellement l'entretien approfondi réalisé par un seul et même interrogateur avec le patient, la vérification des renouvellements des ordonnances et de la ponctualité aux rendez-vous, permettant ainsi en rassemblant un faisceau d'argument de définir des patients ayant une bonne observance thérapeutique de ceux présentant une mauvaise observance. Les facteurs d'observance ont été recherchés grâce à un questionnaire puis les variables continues ont été exprimées en moyenne et les variables discontinues ou discrètes en pourcentage. Une analyse univariée a été réalisée à l'aide d'un test Chi carré pour les variables qualitatives et une analyse des variances ANOVA pour les variables quantitatives. Pour enfin, intégrer toutes les variables significatives ayant un p < 0.05 à l'analyse univariée dans une étude multivariée par régression logistique afin de déterminer les facteurs prédictifs de l'observance.

## Résultats

### Résultats descriptifs

200 patients ont été inclut dans l’étude, le taux d'observance de notre échantillon a avoisiné les 68% avec une prédominance de la tranche d’âge entre 18 et 60 ans et une moyenne à 41 ans. Le taux des femmes constituait 57% de notre échantillon. Le niveau socio économique était estimé moyen à bon chez 55% de l'ensemble des malades avec 45% de ces patients qui rapportent des difficultés d'accès, L'handicape physique et l’éloignement du centre hospitalier universitaire par rapport au centre de la ville. Plus de la moitié de nos patients étaient mariés, avec un taux d'analphabétisme de 44%. 50% des patients étaient professionnellement actifs. La notion de couverture médicale était retrouvée uniquement chez 26% de nos patients. Concernant les caractéristiques cliniques, nous avons noté une prédominance des maladies immunologiques et inflammatoires par rapport aux infectieuses et tumorales. Plus de 70% de notre population d’étude estimaient sa dermatose comme affichante, et rapportaient un retentissement considérable de leur maladie sur leur qualité de vie. 77% de nos patients ont déclaré avoir reçu des explications nettes sur leurs pathologies et bien comprises dans 66% des cas. Sur le plan thérapeutique, les patients ont bénéficié suite à un entretien suffisant selon 97% des malades, d'une prescription médicale jugée non complexe par 72% des concernés. La voie topique était la plus prescrite. La voie orale se retrouve en deuxième position. Pendant la durée de l’étude, les problèmes concernant le traitement soulevées par les malades, intéressaient en premier lieu le coût des médicaments retrouvés chers par plus de la moitié des patients, puis l'utilisation de produits traditionnels chez 14 d'entres eux, et troisièmement la craintes des effets secondaires avec un pourcentage de 7%. Enfin on rapporte l'avis de 95 patients, soit 48% de notre échantillon, sur le rôle explicatif considéré important du pharmacien.

### Résultats de l'analyse univariée

D'un point de vue analytique, l’étude univariée a permis de retenir comme variable statistiquement significative le niveau socio économique et culturel pour les facteurs liés au sujet, le degré de compréhension de la maladie, la visibilité des lésions et l'handicap physique pour les paramètres liés à la maladie, la qualité des explications en ce qui concerne la relation médecin malade et les difficulté d'accès et l'isolement pour les facteurs liées à l'environnement. Par rapport au traitement, les déterminants retenus sont l'efficacité et la simplicité de l'ordonnance délivrée, le cout de la prescription, les effets secondaires des médicaments et de la durée du suivi (Tableau 1). Un taux d'observance non significatif statistiquement mais plus important a été retrouvé chez les femmes, les sujets mariés, les jeunes, les professionnellement actifs, les patients qui possèdent une couverture médicale, sans habitudes toxiques ni maladies associées, et qui n'utilisant pas de traitement traditionnel.

**Tableau 1:** Relation entre la qualité de l'observance et les différents facteurs significatifs

Observance	Bonne	Mauvaise	P
Bon niveau socio économique	79,3%	20,7%	0,0001
Maladie Comprise	79,7	20,3	0.000003
Visibilité importante	79,7%	20,3%	0.01
Présence d'un handicap physique	20,0	80,0	0.01
Maladie bien expliquée	75,2	24,8	0,00005
Nombre moyen de traitement	3,31 + /-1,49	3,98 + /-1,86	0.01
Nombre moyen de traitement oral	1,16 + /-1,43	1,93 + /- 2,18	0.04
Ordonnance simple	75,7%	24,3%	0.00018
Efficacité importante	76,4	23,6	0.01
Absence d'effets secondaires	74,7	25,3	0.00021
Présence de crainte des effets secondaires	35,7	64,3	0.01
Cout du traitement accessible	82,1	17,9	0.0006
Durée moyenne du traitement	13.29 + /-11.17	19.85 + /- 18.83	0.009
Vivre seul	36,0%	64,0%	0.0002
absence de difficultés d'accès	79,1	20,9	0.0002

### Résultats de l'analyse multivariée

L’étude multivariée par régression logistique pour les variables qu'on vient de citer a permit de retenir comme facteurs prédictifs de l'observance: la durée de suivi, l'efficacité et le coût des traitements, l'handicap physique, le degré d'explication de la maladie et les difficultés d'accès. (Tableau 2) En effet, Un bon niveau socio économique est deux fois plus prédictif d'une bonne observance. L'observance est 3,8 fois plus importante si le traitement est efficace, et 4,4 fois meilleure si le coût de traitement n'est pas cher. Une maladie bien expliquée augmente l'observance par un facteur de 3,9. En contre partie, l'handicap physique entrave l'observance avec un facteur de risque de 0,037, auquel se surajoutent les difficultés d'accès aux soins qui dégradent 2,4 fois plus le taux d'observance. La durée de suivi est également un facteur indépendant, elle influence l'observance qui diminue chaque mois par un facteur de 1,027.

**Tableau 2:** Régression logistique des facteurs liés à la mauvaise observance

Facteurs	Odds ratio	Intervalle de confiance [95%]	P
Niveau socio économique	2,333	1,096- 4,966	0,28
Efficacité du traitement	3,849	1,122-13,197	0,74
coût du traitement	4,499	1,536-13,177	0,008
Handicap physique	0,037	0,003- 0,396	0,07
Explication de la maladie	3,995	1,783 -8,952	0,001
Durée de suivi	1,027	1,004-1,051	0,021
Difficultés d'accès aux soins	2,445	1,211-4,569	0,02

## Discussion

L'observance thérapeutique se définit comme la capacité du patient à prendre correctement son traitement, tel qu'il est prescrit par le médecin, c'est une pratique dynamique dans le temps, relativement récente en Dermatologie, qui oscille entre une bonne observance et une non observance sous l'effet de plusieurs facteurs qui peuvent être regroupés en cinq grands chapitres selon qu'ils sont liés au sujet lui-même, à son environnement, à la maladie, au traitement et à la relation médecin malade [1]. À l’échelle internationale, l'Organisation Mondiale de la Santé [2] a opté pour une stratégie qui tend beaucoup plus vers l'optimisation de l'observance thérapeutique que le développement de nouveaux médicaments afin de réduire l'impact d'une mauvaise observance en terme de santé mondiale. Un taux moyen de 50% d'observance médicamenteuse est classiquement retrouvé dans la littérature, notre taux de 62% est proche de celui retrouvé dans la littérature dans d'autres spécialités notamment cardio vasculaires.

### Facteurs déterminants

Les facteurs qui peuvent influencer l'observance sont multiples, ceux retrouvés comme significatifs dans l'analyse multivariée sont: **la durée de suivi:** plus la durée du suivi se prolonge, plus le risque de mauvaise observance augmente, c est ce qu'a montré notre étude, ce résultat est réconforté par l’étude de Carroll qui a établi que l'observance d'un traitement topique diminuait en cas de traitement prolongé, notamment chez les psoriasiques après 8 semaines de traitement [3, 4]. Des études par mesure avec la capsule électronique MEMS (Medication Event Monitoring System) montrent une diminution très claire de l'observance avec le temps après deux mois de suivi, alors que les patients déclarent poursuivre leur traitement de façon identique. Hol. k a objectivé dans son étude européenne qui étudie l'opinion des patients psoriasiques que l'une des principales raisons de non-observance du traitement topique dans le cadre du psoriasis est la lassitude [5]. Cela justifie les visites régulières, d'autant qu'il a été établi que l'observance était meilleure au cours des périodes qui précédaient et qui suivent les consultations, et qu'elle diminue considérablement au bout de 30 jours [6].

**L'efficacité des traitements:** améliore l'observance, ce résultat rejoint celui de l’étude de Fortune DG [7] sur le psoriasis, qui implique la nécessité d’éclaircissements concernant le mode d'action des médicaments prescrits ainsi que la durée approximative de traitement nécessaire pour une réponse thérapeutique favorable.

**Le coût des traitements:** il est surtout engendré par une longue ordonnance ou par les traitements locaux, sachant que la plupart des topiques, et en particulier les émollients ne sont pas remboursables avec pour conséquence un risque de réduction de l'observance. Ce constat est surtout rapporté dans les pays anglo-saxons et en voie de développement [8] tel le cas de notre échantillon où le coût des traitements est un facteur qui entrave l'observance. Cette dernière diminue avec l'augmentation du prix de l'ordonnance.

**Le degré d'explication de la maladie:** est un facteur qui influence l'observance qui est moindre lorsque le malade n'intègre pas l'idée de la chronicité, l’évolution de la maladie et les facteurs aggravants, cet apprentissage dépend du niveau socio-culturel du patient mais surtout de ce qu'on enseigne aux patients et de la durée d'entretien qui lorsqu'elle est suffisante ouvre au patient le champs pour poser des questions, s'exprimer aussi pour permettre au médecin de relever les points encore flou et les expliquer d'avantage. Les difficultés d'accès constituent un obstacle pour le suivi thérapeutique, ceux cités par nos patients ne différent pas de ceux de la littérature à savoir le coût du déplacement, La disponibilité d'un moyen de transport, et l'handicap physique. Notre étude a confirmé certains facteurs connus et a mis le point sur d'autres facteurs peu étudiés tel un traitement traditionnel associé, en effet 14 de nos patients déclarent utiliser un traitement traditionnel en parallèle avec le traitement prescrit. Le taux d'observance chez cette catégorie est très diminué, calculé à 46,2% par rapport à 69,5% chez les patients qui n'utilisent pas de traitement traditionnel associé. Les femmes, les personnes d’âge moyen à avancé, et qui vivent dans les milieux ruraux, sont les plus concernés. Cette pratique représente un coût supplémentaire pour le patient et dégrade la confiance tissée entre malade et médecin. Ce facteur n'a jamais été étudié auparavant mais il doit être pris en considération surtout dans notre contexte et recherché systématiquement lors de l'entretien car les patients qui peuvent dissimuler cette pratique souvent considérée comme une infidélité vis-à-vis de leur médecin. La forme galénique joue également son rôle dans le respect de l'ordonnance, en effet une ordonnance longue et complexe est un facteur significatif de non observance, mais qu'on subdivise cette ordonnance pour connaitre la part de chaque formes galénique, c'est surtout le nombre important de traitements prescrit par voie orale qui est associé à une mauvaise observance de façon significative par rapport à la voie topique et injectable. A travers la revue de la littérature, nous avons constaté que les études réalisées dans notre spécialité sont des études ponctuelles qui s'intéressent à une seule pathologie, alors que la notre se caractérise par sa globalité. Selon nos résultats, sans que cela soit statistiquement significatif, une attention particulière doit être attribuée aux malades suivis pour psoriasis, dermatoses bulleuses, et prurigo qui affichent les taux les plus bas d'observance par rapport aux maladies infectieuses, pelades et dermatose du visage pour lesquelles l'observance est beaucoup plus importante. Le rôle du pharmacien également peu étudié a été souligné par nos patients qui jugeaient son aide importante pour le suivi thérapeutique par 47,5% et modérée par 27,00% d'entre eux.

### Prise en charge

La prise en charge afin d'améliorer l'observance de nos patients consiste en plusieurs étapes dont la plus importante est la phase de l'entretien dans sa forme non directive, qui permet lorsqu'il répond à certains critères à savoir: la bonne réceptivité du malade, la centration sur son vécu et sur le sens qu'il accorde à sa pathologie, de faire la connaissance du patient et de son entourage, et de comprendre ses perceptions, ses motivations, ses conceptions et ses peurs sans induire ses réponses [9–12]. Un tel objectif peut ne pas être acquis dans la première consultation, ce qui implique bien évidement une deuxième le plus proche possible [13]. Ensuite vient l’étape de la prescription qui doit être simple et lisible: on recommande des combinaisons de molécules et des formes retard capables de mieux amortir les oublis, et les irrégularités d'horaire qu'il faut discuter avec le patient à la base de son emploi de temps afin d’éviter les moments de prise contraignants. Le traitement ne doit pas dans la mesure du possible affecter la vie présente du malade: son alimentation, son sommeil et son attention. Il doit répondre à un bon rapport efficacité prix et n'avoir dans le cas échéant que le minimum d'effets secondaires dont il faut informer le malade en l'incitant à consulter pour les premiers symptômes qui le gène. Et devant un risque élevé de mauvaise observance, nous préférons les voies transcutanée et injectable réalisées par un professionnel. Afin de soutenir l'observance de nos patients, particulièrement âgés et adolescents, il faut veiller à l'intégration d'une tierce personne dans la prise en charge en insistant sur le rôle du personnel infirmier qui doit être interpellé au cours de l'hospitalisation pour aider le malade à se familiariser avec son ordonnance. On conseille aux patients qu'ils fassent leurs provisions en médicaments chez le même pharmacien pour créer une relation entre les deux intervenants, qui ne pourrait être que positive pour le suivi thérapeutique Une stratégie de rappel des nombres et heures de prises des différents traitements est essentiel surtout chez les sujets âgés grâce aux piluliers disponibles en forme journalière, hebdomadaire et mensuelle, les horloges programmables et certains types d'appareils téléphoniques [14–16]. Les scènes de simulations du premier contact du malade avec son traitement permettent de déjouer l'anxiété et le stress et de donner au patient une idée sur la posologie à utiliser surtout lorsqu'il s'agit d'un traitement topique [16]. l’éducation thérapeutique trouve sa place particulièrement dans les dermatoses chroniques à fort impact sur la qualité de vie, Il s'agit d'un processus continu, qui comprend des activités organisées d'information, d'apprentissage et d'accompagnement psychosocial concernant la maladie, le traitement et l′ensemble des soins prescrits, qui peut se pratiquer dans notre contexte dans les centres hospitaliers, mais également en compagnes et dans les écoles, visant à rendre le patient aussi autonome que possible dans la gestion da sa vie en compagnie de sa maladie [17, 18]. Ce plan d'action doit viser en particulier: un ancien patient, de bas niveau socio économique, présentant un handicap physique, avec une ordonnance chère, et des difficultés d'accès, victime d’échecs thérapeutiques multiples.

## Conclusion

L'observance thérapeutique dans les maladies chroniques est une notion complexe relativement récente en dermatologie. Cette pratique qui représente un souci à l’échelle internationale, dépend dans notre contexte, de plusieurs facteurs que nous avons déterminés à travers cette étude et qui doivent être ciblé lors de la prise en charge des patients dans l'espoir d'optimiser au maximum notre efficacité thérapeutique.
